# Cost-effectiveness of an occupational therapy-led self-management support programme for multimorbidity in primary care

**DOI:** 10.1093/fampra/cmac006

**Published:** 2022-02-05

**Authors:** Paddy Gillespie, Anna Hobbins, Lynn O’Toole, Deirdre Connolly, Fiona Boland, Susan M Smith

**Affiliations:** Health Economics and Policy Analysis Centre (HEPAC), Institute for Lifecourse & Society (ILAS), CURAM, SFI Research Centre for Medical Devices, NUI Galway, University Road, Galway H91 TK33, Ireland; Health Economics and Policy Analysis Centre (HEPAC), CURAM, SFI Research Centre for Medical Devices, NUI Galway, University Road, Galway H91 TK33, Ireland; Discipline of Occupational Therapy, School of Medicine, Trinity College Dublin, Trinity Centre for Health Sciences, St. James’s Hospital, James’s Street, Dublin 8, D08 NHY1, Ireland; Discipline of Occupational Therapy, School of Medicine, Trinity College Dublin, St. James’s Hospital, James’s Street, Dublin 8, D08 NHY1, Ireland; Data Science Centre and HRB Centre for Primary Care Research, Royal College of Surgeons in Ireland, Mercer Building, Mercer Street Lower, Dublin 2, D02 YN77, Ireland; HRB Centre for Primary Care Research, Department of General Practice, Royal College of Surgeons in Ireland, Mercer Building, Mercer Street Lower, Dublin 2, D02 YN77, Ireland

**Keywords:** complex interventions, cost-effectiveness, multimorbidity

## Abstract

**Background:**

Multimorbidity is a major public health concern. Complex interventions, incorporating individualized care plans, may be appropriate for patients with multimorbidity given their individualized and variable needs. There is a dearth of evidence on the cost-effectiveness of complex multimorbidity interventions.

**Objective:**

This study examines the cost-effectiveness of a 6-week occupational therapy-led self-management support programme (OPTIMAL) for adults with multimorbidity.

**Methods:**

Economic evaluation, from a healthcare perspective, was conducted alongside a randomized controlled trial of 149 adults with multimorbidity. Intervention was the OPTIMAL programme with a comparison of usual primary care. Incremental costs, quality-adjusted life years (QALYs) gained, and expected cost-effectiveness were estimated at 6 months and uncertainty was explored using cost-effectiveness acceptability curves.

**Results:**

The intervention was associated with a mean improvement in QALYs gained of 0.031 per patient (*P*-value: 0.063; 95% confidence intervals [CIs]: −0.002 to 0.063) and a mean reduction in total costs of €2,548 (*P*-value: 0.114; 95% CIs: −5,606 to 509) per patient. At cost-effectiveness threshold values of €20,000 and €45,000 per QALY, the probability of the intervention being cost-effective was estimated to be 0.951 and 0.958, respectively. The results remained consistent across all subgroups examined.

**Conclusions:**

This study adds to the limited evidence base on the cost-effectiveness of complex interventions for multimorbidity, and highlights the potential for the OPTIMAL programme to be cost-effective. Further studies are warranted to explore the clinical and cost-effectiveness of complex interventions for the multimorbidity patient population, and for subgroups within it.

**Trial registration:**

Trial number: ISRCTN67235963.

**Key messages**

- The OPTIMAL programme has the potential to be cost-effective.
- Adds to the limited evidence base on cost-effectiveness for multimorbidity.
- Further economic research on interventions for multimorbidity is necessary.

## Background

Multimorbidity, defined as the presence of 2 or more chronic diseases in the same individual, is a major public health concern, with recent prevalence estimates indicating that in the region of 33.1% of the population may be impacted.^1^ Patients experiencing multiple chronic conditions often have poorer health outcomes, such as poorer physical and mental health functioning,^2^ higher mortality rates,^3^ and higher healthcare costs.^4–6^ From a healthcare planning perspective, the needs of multimorbidity patients are different and guidelines suggest that they be offered individualized care plans, often delivered by a range of healthcare professionals.^1,7,8^ Moreover, while primary care has been highlighted as an ideal setting for such care plans, a 2016 Cochrane review of interventions for multimorbidity and comorbidity in primary care found limited evidence of their benefit or cost-effectiveness.^9^ Reviewers concluded, however, that interventions may be more effective if focused on risk factors common across comorbid conditions or generic outcomes such as daily functioning.^9^ A 2021 systematic review that focused only on multimorbidity included 16 randomized controlled trials (RCTs) and confirms the ongoing lack of clear evidence for cost-effectiveness of multimorbidity interventions.^9^

In this context, occupational therapy (OT) is a discipline with a generic focus on functioning and this focus is also in line with the preferences and priorities of those with multimorbidity.^10,11^ However, there is a paucity of evidence on the clinical and cost-effectiveness of OT interventions for those with multimorbidity.^12,13^ Building on this limited evidence base, the OPTIMAL study employed the Medical Research Council Framework for Complex Interventions^14^ to design, implement, and evaluate an OT-led self-management support programme for adults with multimorbidity in primary care in Ireland.^15^ In brief, the OPTIMAL RCT recruited 149 patients with a diagnosis of multimorbidity. In total, 71 patients were randomized to usual care in the primary care setting and 78 patients were randomized to the intervention. The primary outcome in the clinical effectiveness analysis was health-related quality of life as measured using the EQ-5D-3L instrument.^16^ For the EQ-5D-3L visual analogue scale scores, significant differences were seen between the intervention group and the control group at 3-month follow-up, but no evidence of a difference was found at 6-month follow-up. There was no evidence of differences in the EQ-5D-3L index score and frequency of activity (FAI) participation at 3-month and 6-month follow-up. There were mixed results for secondary outcomes, with significant differences found between the intervention and control group in occupational satisfaction and self-reported hospital outpatient appointments. Furthermore, there was some evidence from subgroup analysis that participants aged <65 years benefitted more from the intervention.^15^ The study suggests that future studies should consider targeting younger adults with multimorbidity (<65 years).^15^

In addition to clinical effectiveness, any decision regarding the adoption of a healthcare intervention in clinical practice should consider its expected cost-effectiveness.^17^ The technique of economic evaluation explores cost-effectiveness by relating the mean difference in cost between alternative treatment options to their mean difference in effectiveness, and by quantifying the uncertainty surrounding these incremental point estimates. This paper reports the cost-effectiveness results from an economic evaluation conducted alongside the RCT to assess an OT-led self-management support programme for multimorbidity in primary care.

## Methods

### Economic evaluation overview

The economic evaluation was conducted following the guidelines for health technology assessment for Ireland.^17^ It consisted of a trial-based analysis with a time horizon of 6 months, the trial follow-up period. The perspective of the healthcare provider was adopted with respect to costing and health outcomes were expressed in terms of quality-adjusted life years (QALYs). Data on resource use and health status were collected via self-report and structured patient questionnaires at baseline and at follow-up. Given the length of follow-up, neither costs nor outcomes were discounted.

The statistical analysis was conducted on an intention-to-treat (ITT) basis. Descriptive statistics, in the form of proportions, means, and standard deviations were estimated for the variables of interest. For the incremental analysis, separate generalized linear model (GLM) regressions were estimated for costs and health outcomes, both of which were estimated controlling for treatment arm and selected baseline covariates. Uncertainty in the analysis was addressed by estimating 95% confidence intervals, hypothesis tests, and cost-effectiveness acceptability curves (CEACs), which link the probability of treatment being cost-effective to a range of potential threshold values (λ) that a health system may be willing to pay per additional QALY gained.^18^ In Ireland, thresholds in the range of €20,000 to €45,000 are generally recommended.^17^ The CEACs were estimated using a nonparametric bootstrapping technique,^19^ which jointly accounts for the correlation in the cost and effect data. In addition, a series of subgroup ((i) aged less than 65 years, (ii) aged 65 years or over, (iii) less than 4 chronic conditions, (iv) 4 or more chronic conditions) and sensitivity analyses, including univariate regression controlling for treatment arm only, were conducted. A complete case analysis was conducted. All analyses were undertaken in the Stata 15 statistical software package.

### Randomized controlled trial

The OPTIMAL study was a pragmatic parallel 2-arm RCT conducted using CONSORT guidelines for the design, conduct, and analysis of RCTs.^20^ The study was approved by the Trinity College Dublin Faculty of Health Sciences Ethics Committee (Ref: 150900) and registered as ISRCTN67235963. The RCT ran from November 2015 to December 2018, with follow-up points at 3 and 6 months. Primary care teams (PCTs) in Ireland typically include family practitioners and practice nurses who are independent contractors, and allied health practitioners, including community nurses, occupational therapists, physiotherapists, dieticians, and social workers, who are employees of the public health system.^20,21^ Trial participants were recruited through PCTs across 8 primary care areas. Clinicians were informed about the study via post or email and presentations at PCT meetings. Referrals by clinicians were forwarded to a study gatekeeper who called patients 7–10 days after referral to seek participation via written informed consent.

Participant inclusion criteria included: aged over 40 years; 2 or more chronic conditions, a minimum of 4 repeat medications, and an ability to travel to attend intervention delivery. As detailed in O’Toole et al.,^15^ the age limit of 40 years was chosen because multimorbidity is relatively uncommon in patients younger than this. Further, a definition of ≥2 chronic conditions was chosen and defined as health problems that require ongoing management over a period of years or decades. The inclusion criteria of ≥4 repeat medications were included to identify a patient group at increased risk of poor health outcomes and more likely to benefit from intervention.^15^ Following baseline data collection, participants were randomized by an independent statistician, using a computer-generated sequence and stratified by gender, into: (i) intervention (OPTIMAL programme) or (ii) control and continued to receive care as usual. Details on the baseline characteristics of the participants are presented in Table 1. One hundred and twenty-one (81%) participants had completed data at 6-month follow-up.

**Table 1.** Baseline characteristics of study participants by treatment arm.

Variable/group	Control		Intervention	
	*N* = 71		*N* = 78	
	Mean	SD	Mean	SD
Number of chronic conditions	4.7	2.1	4.4	1.7
Age (years)	65.9	10.5	65.5	9.3
	Number	%	Number	%
Gender				
Female	50	70.4	53	67.9
Male	21	29.6	25	32.1
Marital status				
In a relationship	0	0	4	5.1
Married	34	47.9	38	48.7
Separated/divorced	14	19.7	11	14.1
Single	16	22.5	8	10.3
Widowed	7	9.9	17	21.8
Education status				
College/university	13	18.3	16	20.5
Primary	27	38.0	29	37.2
Secondary to junior certificate	20	28.2	19	24.4
Secondary to leaving certificate	11	15.5	14	17.9
Employment status				
Carer	2	2.8	1	1.3
Full-time	2	2.8	0	0
Full-time homemaker	1	1.4	3	3.8
Not working due to diagnosis_treatment	23	32.4	17	21.8
Part-time	1	1.4	6	7.7
Retired	37	52.1	46	59.0
Unemployed	5	7.0	5	6.4
Medical card status				
Medical card holder^a^	65	91.5	67	85.9
Mobility aid				
Independent	53	74.6	57	73.1
With aid	17	23.9	15	19.2
Wheelchair user	1	1.4	6	7.7
Living situation				
Living alone	18	25.4	26	33.3
Living with family	53	74.6	49	62.8
Living with others	0	0	3	4

Data were collected during the study period from November 2015 to December 2018.

Medical card status provides the individual with free or subsidized access to primary care services and medications in the Irish healthcare system.

### OPTIMAL intervention

Full details of the OPTIMAL intervention have been previously published.^22,23^ In brief, and as outlined in Table 2, the intervention comprised of a group-based programme, facilitated by occupational therapists with input from physiotherapists and pharmacists, who delivered a series of 2.5-h educational and goal-setting sessions with participants over 6 consecutive weeks in primary or community care centres. Prior to programme delivery, occupational therapists received a half-day training and a facilitator manual to standardize programme delivery and maintain intervention fidelity. A structured curriculum was developed comprising of a set of learning objectives to increase knowledge and skills to manage commonly occurring difficulties identified by individuals with multimorbidity. Topics covered within the 6 weeks included management of fatigue, stress, diet, physical activity, and medication adherence. Goal-setting and action planning is emphasized to facilitate long-term changes to health behaviours. In addition, participants receive a range of materials to support their engagement, including a participant handbook, relaxation CD, information on local resources, and health promotional resources (e.g. exercise booklets, get active your way, healthy eating, mental health).

**Table 2.** Intervention: OPTIMAL programme elements.

Intervention component	OPTIMAL programme
Theory	Self-efficacy theory incorporating influencers including: performance accomplishments, vicarious learning, social/verbal persuasion reinterpretation of physiological and emotional states
Format	Group-based programme Delivered over 6 consecutive weeks; 2.5-h session with tea/coffee break
Location	Primary care centres or community resource centres
Mode of delivery	Educational (includes participant interaction and discussion) and goal-setting components
Facilitators	HSE primary care occupational therapists with input from physiotherapist and pharmacist
Educational component	Week 1: Introduction to self-management, activity, and health and goal-setting Week 2: Fatigue management and health eating Week 3: Maintaining physical activity Week 4: Maintaining mental well-being Week 5: Managing medications Week 6: Communication and programme review
Goal-setting component	Overall programme goals set in week 1 Weekly goal-setting and review
Resources	Participant booklet, relaxation CD, information on local resources, HSE health promotional resources, e.g. exercise booklets, get active your way, healthy eating, information on generics, mental health (https://www.hse.ie/)

HSE, health service executive.

### Cost analysis

Two cost components were included in the analysis, all of which were expressed in Euros (€) in 2019 prices. The first was related to the cost of implementing the intervention in clinical practice. This included a range of resources such as educator and administrator time input, healthcare professional time input (i.e. senior occupational therapists), educational materials and consumables, post, packaging, telephone, and travel expenses. These data were recorded prospectively by the study research team. This cost was allocated to all patients in the intervention arm, as per ITT analysis. Notably, all patients were allocated the same cost irrespective of their level of adherence to the intervention. O’Toole et al.^15^ report that 75.6% of the intervention group attended ≥3 intervention sessions, which was the definition of adherence adopted.

Second, costs relating to the use of primary and secondary healthcare services over the course of the trial were estimated for individuals in both treatment arms. This included the costs of general practitioner, outpatient, accident and emergency visits, and hospital admissions. Resource use was captured via self-report questionnaires at baseline and follow-up and for a period of 6 months. A vector of unit costs was applied to calculate the cost associated with each resource activity at each time point. Unit cost estimates for each activity were based on national data sources and, where necessary, were transformed to Euros (€) in 2019 prices using appropriate indices^24^ (see Table 3). In sensitivity analysis, the effects of inflating the intervention unit cost inputs by 10% and 50% were examined. Moreover, the effect of a minimum intervention cost of €182, based on the intervention being delivered by staff-grade occupational therapists, was considered.

**Table 3.** Categories of unit cost estimates in 2019 (€) prices.

Resource item	Activity	Unit cost €	Source
Healthcare resources			
GP visits	Per visit	€60	Study records
Outpatient visits	Per visit public	€136	HPO
Inpatient days	Per inpatient night	€933	HPO
A&E visits	Per visit	€268	HPO

GP, general practitioner.

(HPO) Healthcare Pricing Office Admitted Price List 2019. Unit costs in 2019 prices. Where necessary unit costs were inflated using the health component of the consumer price index from the Central Statistics office.^24^

For the purposes of the incremental analysis, a total cost at 6 months follow-up variable was constructed. The individual resource costs were summed to compute the total cost variable. Estimation of incremental total cost was undertaken using a GLM regression, controlling for treatment arm and baseline total cost, and assuming a Gamma variance function and log link function.^25^

### Effectiveness analysis

Health outcomes in the analysis were expressed in terms of QALYs gained, calculated using the EuroQol EQ-5D-3L instrument,^16^ a standardized tool designed to describe and value health status. The EQ-5D-3L consists of 5 dimensions: mobility, self-care, usual activities, pain or discomfort, and anxiety or depression; and each dimension has 3 levels of severity: no problems, moderate problems, or extreme problems. A scoring algorithm is applied to transform EQ-5D responses into a single health state index score, which typically range from 0 (equivalent to death) to 1 (equivalent to good health), although a small number of health states are valued as worse than death. The scoring algorithm is based on values elicited via a time trade-off approach for the UK population.^26^ Quality-adjusted life expectancy over a period of time is calculated by weighting each component of the time period by its relevant health state index score, using the area under the curve method.^27^ For the purposes of the incremental analysis, a QALYs gained at 6 months variable was constructed using the EQ-5D scores for each participant at baseline, 3 and 6 months using the area under the curve method. Estimation of incremental QALYs gained was undertaken using a GLM regression, controlling for treatment arm and baseline EQ-5D score, and assuming a Gaussian variance function and identity link function.

## Results

Raw data estimates for resource use, costs, and EQ-5D index scores and QALYs are presented in Table 4. Details on missing data are also presented, which were deemed not to be systematically different across the treatment arms. The results from the incremental cost-effectiveness analysis are presented in Table 5. Results from the subgroup and sensitivity analyses are presented in the Supplementary Appendix.

**Table 4.** Resource use, costs, EQ-5D scores, and QALY estimates at baseline and follow-up.

Resource items	Intervention		Control		Intervention		Control	
	Baseline—for the 6-month period prior to randomization Mean (SD)				Follow-up—for the 6-month period post-randomization Mean (SD)			
	Usage	Cost€	Usage	Cost€	Usage	Cost€	Usage	Cost €
GP visits	5.68 (4.58)	341 (275)	5.66 (5.87)	340 (352)	4.31 (3.77)	259 (227)	4.67 (3.33)	280 (200)
Outpatient visits	4.37 (6.14)	594 (835)	5.10 (9.65)	693 (1,312)	2.38 (2.54)	323 (346)	4.09 (3.12)	556 (424)
Inpatient nights	3.60 (11.34)	3,361 (10,581)	0.96 (2.81)	893 (2,622)	1.59 (5.50)	1,487 (5,137)	3.79 (9.82)	3,535 (9,161)
A&E visits	0.81 (1.79)	216 (481)	0.44 (0.69)	117 (185)	0.55 (1.31)	146 (351)	0.47 (0.95)	127 (254)
Total healthcare cost		4,513 (10,672)		2,044 (3,564)		2,215 (5,329)		4,499 (9,258)
Optimal programme						402 (0)		0 (0)
Total cost						2,617 (5,329)		4,499 (9,258)
Health outcomes								
EQ-5D-3L score—baseline					0.44 (0.35)		0.40 (0.36)	
EQ-5D-3L score—follow-up—3 months					0.50 (0.34)		0.42 (0.37)	
EQ-5D-3L score—follow-up—6 months					0.51 (0.34)		0.35 (0.38)	
QALYs gained					0.25 (0.16)		0.20 (0.16)	

A&E, accident and emergency; GP, general practitioner.

Completeness of data: Intervention: *Baseline*—0% missing data for GP visits, outpatient visits, hospital inpatient nights, A&E visits, and EQ-5D. Intervention: *Follow-up—*18% missing data on GP visits, 18% for outpatient visits, 18% for days in hospital inpatient nights, 18% for A&E visits, 18% for total cost, 7% for EQ-5D.2, 18% for EQ-5D.3, and 21% for QALYs. Control: *Baseline*—0% missing data for GP visits, outpatient visits, hospital inpatient nights, A&E visits, and EQ-5D. Control: *Follow-up—*20% missing data on GP visits, 20% for outpatient visits, 20% for days in hospital inpatient nights, 20% for A&E visits, 20% for total cost, 16% for EQ-5D.2, 20% for EQ-5D.3, and 28% for QALYs. Data were collected during the study period from November 2015 to December 2018.

**Table 5.** Incremental cost-effectiveness results.

Variable/analysis		Incremental analysis (intervention minus control)			
Cost analysis					
Difference in mean total cost € (95% CIs) [*P*-value]		−2,548 (−5,606, 509) [0.114]			
Effectiveness analysis					
Difference in mean QALYs (95% CI) [*P*-value]		0.031 (−0.002, 0.063) [0.063]			
Probability (%) that the intervention is cost-effective for threshold value (λ)					
λ = €0	λ = €5,000	λ = €10,000	λ = €20,000	λ = €30,000	λ = €45,000
0.941	0.945	0.948	0.951	0.955	0.958

Cost analyses: GLM regression model, with log link function, Gamma variance function, estimated controlling for *treatment group* and *baseline cost*. QALYs analyses: GLM regression model, with iden link function, Gaussian variance function, estimated controlling for *treatment group* and *baseline EQ-5D-3L*. Expected cost-effectiveness analysis: probabilities estimated using nonparametric bootstrapping technique based on 1,000 bootstrapped resamples. Data were collected during the study period from November 2015 to December 2018.

The total cost of implementing the intervention was €31,321, giving a mean cost per participant estimate of €402. The intervention was, on average, less costly and more effective than usual care. With respect to total costs at 6 months, the mean cost per patient estimates were €2,617 for the intervention arm and €4,499 for control arm. The intervention was associated with a reduction in mean costs of €2,548 (*P*-value: 0.114; 95% CIs: −€5606 to €509) per patient. In terms of QALYs gained at 6 months, the mean estimates were 0.25 for the intervention and 0.20 for the control. The intervention was associated with an increase in mean QALYs of 0.031 (*P*-value: 0.063; 95% CI: −0.002 to 0.063) per patient. The expected cost-effectiveness results indicate that at alternative threshold values of €5,000, €10,000, €20,000, €30,000, and €45,000, the probability of the intervention being cost-effective was estimated to be 0.945, 0.948, 0.951, 0.955, and 0.958, respectively.

The results from a series of subgroup and sensitivity analyses are presented in the Supplementary Appendix, and broadly reflect those from the base-case analysis. With respect to the subgroup analyses there was a higher probability of the intervention being cost-effective at each threshold value for those under 65 years compared to over 65 years, and for those with 4 or more conditions compared to those with fewer conditions.

## Discussion

A trial-based economic evaluation was undertaken to assess the cost-effectiveness of an OT-led self-management support intervention for adults with multimorbidity in a primary care setting. The OPTIMAL intervention appeared to be cost-effective relative to usual care at 6 months follow-up for the full patient population, and potentially even more so, for particular patient subgroups. Notably, the estimated probability of the intervention being cost-effective remained appreciably higher than 90% across the range of cost-effectiveness threshold values that are adopted in Ireland.

Uncertainty in the analysis was also characterized using standard statistical inference methods, which indicated no statistically significant differences in mean costs and mean QALYs across treatment arms, and corresponds to the findings from the clinical study. Indeed, our findings point to a common issue that arises in trial-based economic evaluation; that is, the question of whether observed nonsignificance in economics results arises from the study being statistically underpowered to detect meaningful differences in these outcomes.^28,29^ To address this problem, the weight of cost-effectiveness evidence in favour of the intervention relative to the control is presented using CEACs, rather than relying solely on showing significance at conventional levels.^28,29^ Moreover, this approach explores the joint uncertainty in the incremental cost and incremental QALY estimates, and translates this into a single estimate of expected cost-effectiveness in order to aid the decision-making process.

Importantly, the estimated reduction in mean costs for the intervention relative to the control over the course of the trial was the main driver in the observed cost-effectiveness results, with the greater levels of uncertainty surrounding the estimate for the difference in mean QALYs. Notably, the cost savings arising from observed differences in utilization of primary and secondary care services offset the additional implementation costs of the intervention, leading to an overall reduction in costs relative to usual care. While challenging to pinpoint the exact mechanism at play, it may be the case that more proactive approaches to chronic disease management delivered in the primary care setting go to reduce the need for more expensive hospital care. It is ultimately the remit of decision-makers to determine whether the level of certainty with which these results are estimated is sufficient to justify the adoption of the OPTIMAL intervention in clinical practice. In doing so, decision-makers would need to carefully consider the balance of the cost and effect contributions to the overall cost-effectiveness result.

This study adds to the limited international evidence base on the cost-effectiveness of complex interventions for patient populations with multimorbidity or comorbidity, as detailed in the 2016 Cochrane review of interventions in primary care. For example, a trial-based economic evaluation of a multicondition collaborative treatment programme delivered in primary care was found to be cost-effective for patients with depression and poorly controlled diabetes or coronary heart disease.^30^ However, a more recent study of a patient-centred approach to managing multimorbidity in primary care was only found to have a 50.8% probability of being cost-effective at a threshold value of £20,000.^31^ Conversely, in an evaluation of CARE Plus, a whole-system primary care-based complex multimorbidity intervention, a cost-effectiveness probability of 0.93 was reported for a threshold of £30,000 per QALY.^32^ Our results further highlight the potential for primary care-based interventions to be cost-effective for patient populations with multimorbidity or comorbidity. From an Irish perspective, where the approach to chronic disease management in primary care has been reactive in nature, our findings highlight the potential resource implications of adopting a more proactive and systematic approach to care for people with 1 or more chronic conditions. This is particularly relevant given future projections of a growing number of people with chronic disease and concerns over the ability of an already resource constrained healthcare infrastructure to cope with the expected increase in need. Notably, the observed resource usage patterns in this study appear to be true for chronic conditions, including type 1 diabetes,^33^ type 2 diabetes,^34^ and heart disease.^35^ While further evidence is required, the results suggest a tentative pattern of beneficial resource implications from more proactive multidisciplinary approaches to chronic disease management in Irish primary care.

There are a number of limitations in the analysis. First, the limitations relating to the conduct of the RCT, as outlined in the main trial publication,^15^ also apply to the economic evaluation, specifically the use of self-report data for healthcare utilization and the use of multiple assessors for baseline data collection. Relatedly, missing data were a factor in the analysis, but patterns did not differ across treatment arms. Further, the inclusion criteria, while pragmatically selected, may be deemed somewhat restrictive and thereby, limit the generalizability of the findings. Second, the time horizon of the economic evaluation was limited to the trial follow-up period of 6 months; thereby excluding costs and benefits that arise beyond 6 months and over the remainder of the patients’ lifetime. This may be particularly relevant in the context of chronic disease, for which short-term interventions may have long-term implications. The issue of transferability of data from external sources to the Irish setting is an important consideration. In particular, in the absence of an EQ-5D-3L value set for the Irish population, utility weights for the UK population were employed.

While the cost analysis was conducted from the health service perspective and included a range of resource use activities, certain resource items which may have impacted our findings were not captured. For example, costs of medications, tests, procedures, and community care were not calculated for pragmatic and logistical reasons. Moreover, future studies should consider a wider societal perspective, and capture, for example, private patient costs, productivity losses, and informal care costs. Further, the ITT approach to costing the intervention may be questioned, as only 75.6% of participants were fully adherent. Notably, separate regressions were adopted for the analysis of the total cost and QALYs variables. While simultaneous regression approaches may be superior in accounting for correlation, the approach adopted was chosen to explicitly account for the distributional nature of the cost data. Finally, the process of conducting cost analysis in Ireland is also compromised by the lack of nationally available unit cost data. In estimating unit costs for individual resource activities, we endeavoured at all times to be conservative in any assumptions adopted.

In conclusion, our results suggest that the OPTIMAL multimorbidity programme has the potential to be cost-effective compared to usual care. Further research on the clinical and cost-effectiveness of OT-led interventions targeting the multimorbidity patient population, and subgroups within it, are required to build the evidence base in this context.

## Supplementary Material

cmac006_suppl_Supplementary_Appendix

cmac006_suppl_Supplementary_Checklist

## Data Availability

The data from this study will be made available on an open access repository, please contact the corresponding author if you have any queries regarding the supporting data in the interim.

## References

[1] Nguyen H , Manolova G, Daskalopoulou C, Vitoratou S, Prince M, Prina AM. Prevalence of multimorbidity in community settings: a systematic review and meta-analysis of observational studies. J Comorb. 2019;9:2235042X19870934. doi:10.1177/2235042X19870934. PMID: 31489279; PMCID: PMC6710708. PMC6710708 31489279

[2] Ryan A , Wallace E, O’Hara P, Smith SM. Multimorbidity and functional decline in community-dwelling adults: a systematic review. Health Qual Life Outcomes. 2015;13:168. doi:10.1186/s12955-015-0355-9. PMID: 26467295; PMCID: PMC4606907. 26467295 PMC4606907

[3] Nunes BP , Flores TR, Mielke GI, Thumé E, Facchini LA. Multimorbidity and mortality in older adults: a systematic review and meta-analysis. Arch Gerontol Geriatr. 2016;67:130–138. doi:10.1016/j.archger.2016.07.008. PMID: 27500661. 27500661

[4] Xu X , Mishra GD, Jones M. Evidence on multimorbidity from definition to intervention: an overview of systematic reviews. Ageing Res Rev. 2017;37:53–68. doi:10.1016/j.arr.2017.05.003. PMID: 28511964. 28511964

[5] Marengoni A , Angleman S, Melis R, Mangialasche F, Karp A, Garmen A, Meinow B, Fratiglioni L. Aging with multimorbidity: a systematic review of the literature. Ageing Res Rev. 2011;10(4):430–439. 21402176 10.1016/j.arr.2011.03.003

[6] Glynn LG , Valderas JM, Healy P, Burke E, Newell J, Gillespie P, Murphy AW. The prevalence of multimorbidity in primary care and its effect on health care utilization and cost. Fam Pract. 2011;28(5):516–523. 21436204 10.1093/fampra/cmr013

[7] Academy of Medical Sciences. Multimorbidity: a priority for global health research. 2018 [accessed 2020 Dec 10]. https://acmedsci.ac.uk/policy/policy-projects/multimorbidity.

[8] Salisbury C , Johnson L, Purdy S, Valderas JM, Montgomery AA. Epidemiology and impact of multimorbidity in primary care: a retrospective cohort study. Br J Gen Pract. 2011;61(582):e12–e21. 21401985 10.3399/bjgp11X548929 PMC3020068

[9] Smith SM , Wallace E, O’Dowd T, Fortin M. Interventions for improving outcomes in patients with multimorbidity in primary care and community settings. Cochrane Database Syst Rev. 2016;3(3):Cd006560. doi:10.1002/14651858.CD006560.pub3. PMID: 26976529; PMCID: PMC6703144. 26976529 PMC6703144

[10] Noël PH , Frueh BC, Larme AC, Pugh JA. Collaborative care needs and preferences of primary care patients with multimorbidity. Health Expect. 2005;8(1):54–63. 15713171 10.1111/j.1369-7625.2004.00312.x PMC5060269

[11] Bratzke LC , Muehrer RJ, Kehl KA, Lee KS, Ward EC, Kwekkeboom KL. Self-management priority setting and decision-making in adults with multimorbidity: a narrative review of literature. Int J Nurs Stud. 2015;52(3):744–755. 25468131 10.1016/j.ijnurstu.2014.10.010 PMC4315694

[12] Leland NE , Fogelberg DJ, Halle AD, Mroz TM. Occupational therapy and management of multiple chronic conditions in the context of health care reform. Am J Occup Ther. 2016;71(1):7101090010p7101090011–7101090010p7101090016. 10.5014/ajot.2017.711001 PMC5182013 28027031

[13] Hand CL , Letts LJ, von Zweck CM. An agenda for occupational therapy’s contribution to collaborative chronic disease research. Can J Occup Ther. 2011;78(3):147–155. 21699008 10.2182/cjot.2011.78.3.2

[14] Craig P , Dieppe P, Macintyre S, Michie S, Nazareth I, Petticrew M. Developing and evaluating complex interventions: the new Medical Research Council guidance. *BMJ* . 2008;337:a1655. doi:10.1136/bmj.a1655. PMC2769032 18824488

[15] O’Toole L , Connolly D, Boland F, Smith S. Enhancing self-management of multimorbidity in primary care: a randomised controlled trial. Br J Gen Pract. 2021;71(9):bjgp20X714185. doi:10.3399/bjgp20X714185. PMC7959668 33685920

[16] EuroQol Research Foundation. EQ-5D-3L user guide: basic information on how to use the EQ-5D-3L instrument. 2015 [accessed 2021 Mar 1]. https://euroqol.org/publications/userguides.

[17] H.I.Q.A. Guidelines for the economic evaluation of health technologies in Ireland. 2020 [accessed 2021 Jan 28]. https://www.hiqa.ie/sites/default/files/2020-09/HTA-Economic-Guidelines-2020.pdf.

[18] Briggs A , Drummond MF, McGuire A. Handling uncertainty in economic evaluation and presenting the results. Economic evaluation in health care: merging theory with practice. New York: Oxford University Press; 2001.

[19] Fenwick E , Claxton K, Sculpher M. Representing uncertainty: the role of cost-effectiveness acceptability curves. Health Econ. 2001;10(8):779–787. 11747057 10.1002/hec.635

[20] Schulz KF , Altman DG, Moher D, Group C. CONSORT 2010 statement: updated guidelines for reporting parallel group randomised trials. Trials. 2010;11(1):32. 21350618 10.4103/0976-500X.72352 PMC3043330

[21] Tierney E , O’Sullivan M, Hickey L, Hannigan A, May C, Cullen W, Kennedy N, Kineen L, MacFarlane A. Do primary care professionals agree about progress with implementation of primary care teams: results from a cross sectional study. BMC Fam Pract. 2016;17(1):163. 27875994 10.1186/s12875-016-0541-9 PMC5120534

[22] Garvey J , Connolly D, Boland F, Smith SM. OPTIMAL, an occupational therapy led self-management support programme for people with multimorbidity in primary care: a randomized controlled trial. BMC Fam Pract. 2015;16(1):59. 25962515 10.1186/s12875-015-0267-0 PMC4438474

[23] Toole LO , Connolly D, Smith S. Impact of an occupation-based self-management programme on chronic disease management. Aust Occup Ther J. 2013;60(1):30–38. 23414187 10.1111/1440-1630.12008

[24] C.S.O. Consumer price index by commodity group, month and statistic. CPMO1. 2019 [accessed 2019 Aug 5]. https://www.cso.ie/px/pxeirestat/Statire/SelectVarVal/Define.asp?maintable=CPM01&PLanguage=0.

[25] Drummond MF , Sculpher MJ, Claxton K, Stoddart GL, Torrance GW. Methods for the economic evaluation of health care programmes. 4th ed. Oxford (UK): Oxford University Press; 2015.

[26] Dolan P , Gudex C, Kind P, Williams A. The time trade-off method: results from a general population study. Health Econ. 1996;5(2):141–154. doi:10.1002/(SICI)1099-1050(199603)5:2<141::AID-HEC189>3.0.CO;2-N. PMID: 8733106. 8733106

[27] Orenstein DM , Kaplan RM. Measuring the quality of well-being in cystic fibrosis and lung transplantation. The importance of the area under the curve. Chest. 1991;100(4):1016–1018. 1914549 10.1378/chest.100.4.1016

[28] Gomes M , Ng ESW, Grieve R, Nixon R, Carpenter J, Thompson SG. Developing appropriate methods for cost-effectiveness analysis of cluster randomized trials. Med Decis Making. 2011;32(2):350–361. 22016450 10.1177/0272989X11418372 PMC3757919

[29] Briggs AH. A Bayesian approach to stochastic cost-effectiveness analysis: an illustration and application to blood pressure control in type 2 diabetes. Int J Technol Assess Health Care. 2001;17(1):69–82. 11329846 10.1017/s0266462301104071

[30] Katon W , Russo J, Lin EH, Schmittdiel J, Ciechanowski P, Ludman E, Peterson D, Young B, Von Korff M. Cost-effectiveness of a multicondition collaborative care intervention: a randomized controlled trial. Arch Gen Psychiatry. 2012;69(5):506–514. 22566583 10.1001/archgenpsychiatry.2011.1548 PMC3840955

[31] Thorn J , Man M-S, Chaplin K, Bower P, Gaunt D, Fitzpatrick B, Gardner C, Guthrie B, Hollinghurst S, Lee V, et al. Cost-effectiveness of a patient-centred approach to managing multimorbidity in primary care: a pragmatic cluster randomised controlled trial. BMJ Open. 2020;10(1):e030110. 10.1136/bmjopen-2019-030110 PMC7044971 31959601

[32] Mercer SW , Fitzpatrick B, Guthrie B, Fenwick E, Grieve E, Lawson K, Boyer N, McConnachie A, Llyod SM, O’Brien R, et al. The CARE Plus study—a whole-system intervention to improve quality of life of primary care patients with multimorbidity in areas of high socioeconomic deprivation: exploratory cluster randomised controlled trial and cost-utility analysis. BMC Med. 2016;14:88. doi:10.1186/s12916-016-0634-2. 27328975 PMC4916534

[33] Gillespie P , O’Shea E, O’Hara M, Dinneen SF. Cost effectiveness of group follow-up after structured education for type 1 diabetes: a cluster randomised controlled trial. Trials. 2014;15:1–11. 24927851 10.1186/1745-6215-15-227 PMC4070096

[34] Gillespie P , O’Shea E, Paul G, O’Dowd T, Smith SM. Cost effectiveness of peer support for type 2 diabetes. Int J Technol Assess Health Care. 2012;28(1):3–11. 22617733 10.1017/S0266462311000663

[35] Gillespie P , O’Shea E, Murphy AW, Byrne MC, Byrne M, Smith SM, Cupples ME. The cost-effectiveness of the SPHERE intervention for the secondary prevention of coronary heart disease. Int J Technol Assess Health Care. 2010;26(3):263–271. 20584354 10.1017/S0266462310000358

