# Supplementary material for: Cost-effectiveness of an occupational therapy-led self-management support programme for multimorbidity in primary care
Source: Fam Pract. 2022 Feb 5;39(5):826–33. doi: 10.1093/fampra/cmac006 (PMC9508868; doi:10.1093/fampra/cmac006)
Supplement: cmac006_suppl_Supplementary_Appendix [file cmac006_suppl_supplementary_appendix.docx]

**APPENDIX**

**Table A1 – OPTIMAL Intervention Costs**

| **Resource item** | **Total Cost** | **Total Cost Per**  **Patient** |
| --- | --- | --- |
| **Facilitator Training**  Trainer, Healthcare Professional Time Input; Educational Materials and Consumables; Venue Rental & Refreshments; Travel Expenses; Phone Calls, Postage &Packaging  **Patient Recruitment**  Healthcare Professional Time Input; Phone Calls, Postage &Packaging  **Group-Based Sessions**  Healthcare Professional Time Input; Patient Notification, Meeting Organisation  Educational Materials and Consumables; Handbooks and Resource Packs; Travel Expenses; Phone Calls, Postage and Packaging | €615  €1,400  €24,676  €4,630.20 | €7.89  €17.95  €316.35  €59.36 |
| **Intervention Cost (Base-case Analysis)** | **€31,321.16** | **€401.55** |
| Sensitivity Analysis 1 + 10% | €34,453.28 | €441.70 |
| Sensitivity Analysis 2 + 50% | €46,981.74 | €602.33 |
| Sensitivity Analysis 3 – Low cost | €14,218.02 | €182.28 |

**Table A2 – Subgroup Analysis**

| **Variable/ Analysis** | | | **Subgroup Analysis: Aged 65 Years and Over (n=82)** | | | |
| --- | --- | --- | --- | --- | --- | --- |
| **Cost Analysis** | | |  | | | |
| Difference in Mean Total Cost €  (95% CIs) [p-value] | | | **-3457**  **(-8638, 1724) [0.191** | | | |
| **Effectiveness Analysis** | | |  | | | |
| Difference in Mean QALYs  (95% CIs) [p-value] | | | 0.026  (-0.014, 0.066) [0.205] | | | |
|  | | | | | | |
| ***Probability (%) that the Intervention is Cost Effective for Threshold Value (λ)*** | | | | | | |
| **λ = €0** | **λ = €5,000** | **λ = €10,000** | | **λ = €20,000** | **λ = €30,000** | **λ = €45,000** |
| 0.849 | 0.853 | 0.861 | | 0.875 | 0.890 | 0.958 |
| **Variable/ Analysis** | | | **Subgroup Analysis: Aged Under 65 Years (n=67)** | | | |
| **Cost Analysis** | | |  | | | |
| Difference in Mean Total Cost €  (95% CIs) [p-value] | | | **-1324**  **(-4372,1725) [0.395]** | | | |
| **Effectiveness Analysis** | | |  | | | |
| Difference in Mean QALYs  (95% CIs) [p-value] | | | 0.035  (-0.021, 0.092) [0.222] | | | |
|  | | | | | | |
| ***Probability (%) that the Intervention is Cost Effective for Threshold Value (λ)*** | | | | | | |
| **λ = €0** | **λ = €5,000** | **λ = €10,000** | | **λ = €20,000** | **λ = €30,000** | **λ = €45,000** |
| 0.768 | 0.807 | 0.826 | | 0.860 | 0.884 | 0.896 |
| **Variable/ Analysis** | | | **Subgroup Analysis: 4 or More Chronic Conditions (n=96)** | | | |
| **Cost Analysis** | | |  | | | |
| Difference in Mean Total Cost €  (95% CIs) [p-value] | | | **-3527**  **(-9291, 2237) [0.230]** | | | |
| **Effectiveness Analysis** | | |  | | | |
| Difference in Mean QALYs  (95% CIs) [p-value] | | | 0.045  (0.005, 0.085) [0.029] | | | |
|  | | | | | | |
| ***Probability (%) that the Intervention is Cost Effective for Threshold Value (λ)*** | | | | | | |
| **λ = €0** | **λ = €5,000** | **λ = €10,000** | | **λ = €20,000** | **λ = €30,000** | **λ = €45,000** |
| 0.897 | 0.907 | 0.921 | | 0.947 | 0.957 | 0.963 |
| **Variable/ Analysis** | | | **Subgroup Analysis: < 4 Chronic Conditions (n=53)** | | | |
| **Cost Analysis** | | |  | | | |
| Difference in Mean Total Cost €  (95% CIs) [p-value] | | | **-1712**  **(-5008, 1584) [0.309]** | | | |
| **Effectiveness Analysis** | | |  | | | |
| Difference in Mean QALYs  (95% CIs) [p-value] | | | 0.002  (-0.053, 0.56) [0.956] | | | |
|  | | | | | | |
| ***Probability (%) that the Intervention is Cost Effective for Threshold Value (λ)*** | | | | | | |
| **λ = €0** | **λ = €5,000** | **λ = €10,000** | | **λ = €20,000** | **λ = €30,000** | **λ = €45,000** |
| 0.536 | 0.552 | 0.560 | | 0.584 | 0.607 | 0.637 |

**Cost Analyses**: GLM regression model, with log link function, Gamma variance function, estimated controlling for ***treatment group*** and ***baseline cost***

**QALYs Analyses**: GLM regression model, with identity link function, Gaussian variance function, estimated controlling for ***treatment group*** and ***baseline EQ-5D-3L***

**Expected Cost Effectiveness Analysis**: Probabilities estimated using nonparametric bootstrapping technique based on 1000 bootstrapped resamples.

**Note:** Data were collected during the study period from November 2015 to December 2018

**Table A3 – Sensitivity Analysis - Intervention Cost Variations**

| **Variable/ Analysis** | | | **Sensitivity Analysis –OPTIMAL Intervention Cost Increased by 10%** | | | |
| --- | --- | --- | --- | --- | --- | --- |
| **Cost Analysis** | | |  | | | |
| Difference in Mean Total Cost €  (95% CIs) [p-value] | | | **-2495**  **(-5613, 623) [0.117]** | | | |
| **Effectiveness Analysis** | | |  | | | |
| Difference in Mean QALYs  (95% CIs) [p-value] | | | 0.031  (-0.002, 0.063) [0.063] | | | |
|  | | | | | | |
| ***Probability (%) that the Intervention is Cost Effective for Threshold Value (λ)*** | | | | | | |
| **λ = €0** | **λ = €5,000** | **λ = €10,000** | | **λ = €20,000** | **λ = €30,000** | **λ = €45,000** |
| 0.938 | 0.945 | 0.947 | | 0.950 | 0.954 | 0.958 |
| **Variable/ Analysis** | | | **Sensitivity Analysis –OPTIMAL Intervention Cost Increased by 50%** | | | |
| **Cost Analysis** | | |  | | | |
| Difference in Mean Total Cost €  (95% CIs) [p-value] | | | **-2289**  **(-5271, 693) [0.133]** | | | |
| **Effectiveness Analysis** | | |  | | | |
| Difference in Mean QALYs  (95% CIs) [p-value] | | | 0.031  (-0.002, 0.063) [0.063] | | | |
|  | | | | | | |
| ***Probability (%) that the Intervention is Cost Effective for Threshold Value (λ)*** | | | | | | |
| **λ = €0** | **λ = €5,000** | **λ = €10,000** | | **λ = €20,000** | **λ = €30,000** | **λ = €45,000** |
| 0.931 | 0.938 | 0.945 | | 0.950 | 0.952 | 0.957 |
| **Variable/ Analysis** | | | **Sensitivity Analysis – Cost of OPTIMAL Intervention at low value of €182** | | | |
| **Cost Analysis** | | |  | | | |
| Difference in Mean Total Cost €  (95% CIs) [p-value] | | | **-2853**  **(-6287, 582) [0.104]** | | | |
| **Effectiveness Analysis** | | |  | | | |
| Difference in Mean QALYs  (95% CI) [p-value] | | | 0.031  (-0.002, 0.063) [0.063] | | | |
|  | | | | | | |
| ***Probability (%) that the Intervention is Cost Effective for Threshold Value (λ)*** | | | | | | |
| **λ = €0** | **λ = €5,000** | **λ = €10,000** | | **λ = €20,000** | **λ = €30,000** | **λ = €45,000** |
| 0.946 | 0.950 | 0.953 | | 0.953 | 0.958 | 0.959 |

**Cost Analyses**: GLM regression model, with log link function, Gamma variance function, estimated controlling for ***treatment group*** and ***baseline cost***

**QALYs Analyses**: GLM regression model, with identity link function, Gaussian variance function, estimated controlling for ***treatment group*** and ***baseline EQ-5D-3L***

**Expected Cost Effectiveness Analysis**: Probabilities estimated using nonparametric bootstrapping technique based on 1000 bootstrapped resamples.

**Note:** Data were collected during the study period from November 2015 to December 2018

**Table A4 – Sensitivity Analysis – Univariate Regression Results**

| **Variable/ Analysis** | | | **Base-case Analysis** | | | |
| --- | --- | --- | --- | --- | --- | --- |
| **Cost Analysis** | | |  | | | |
| Difference in Mean Total Cost €  (95% CIs) [p-value] | | | **-1881**  **(-4655, 892) [0.1811]** | | | |
| **Effectiveness Analysis** | | |  | | | |
| Difference in Mean QALYs  (95% CI) [p-value] | | | 0.042  (-0.017, 0.101) [0.163] | | | |
|  | | | | | | |
| ***Probability (%) that the Intervention is Cost Effective for Threshold Value (λ)*** | | | | | | |
| **λ = €0** | **λ = €5,000** | **λ = €10,000** | | **λ = €20,000** | **λ = €30,000** | **λ = €45,000** |
| 0.873 | 0.889 | 0.896 | | 0.904 | 0.910 | 0.923 |
| **Variable/ Analysis** | | | **Subgroup Analysis: Aged 65 Years and Over (n=82)**) | | | |
| **Cost Analysis** | | |  | | | |
| Difference in Mean Total Cost €  (95% CIs) [p-value] | | | **-2255**  **(-6674, 2165) [0.3103]** | | | |
| **Effectiveness Analysis** | | |  | | | |
| Difference in Mean QALYs  (95% CIs) [p-value] | | | 0.016  (-0.048, 0.079)[0.623] | | | |
|  | | | | | | |
| ***Probability (%) that the Intervention is Cost Effective for Threshold Value (λ)*** | | | | | | |
| **λ = €0** | **λ = €5,000** | **λ = €10,000** | | **λ = €20,000** | **λ = €30,000** | **λ = €45,000** |
| 0.675 | 0.692 | 0.701 | | 0.727 | 0.744 | 0.769 |
| **Variable/ Analysis** | | | **Subgroup Analysis: Aged Under 65 Years (n=67)**) | | | |
| **Cost Analysis** | | |  | | | |
| Difference in Mean Total Cost €  (95% CIs) [p-value] | | | **-1368**  **(-4227, 1490) [0.3389]** | | | |
| **Effectiveness Analysis** | | |  | | | |
| Difference in Mean QALYs  (95% CIs) [p-value] | | | 0.081  (-0.014, 0.177) [0.092] | | | |
|  | | | | | | |
| ***Probability (%) that the Intervention is Cost Effective for Threshold Value (λ)*** | | | | | | |
| **λ = €0** | **λ = €5,000** | **λ = €10,000** | | **λ = €20,000** | **λ = €30,000** | **λ = €45,000** |
| 0.866 | 0.898 | 0.930 | | 0.952 | 0.964 | 0.968 |
| **Variable/ Analysis** | | | **Subgroup Analysis: 4 or More Chronic Conditions (n=96)** | | | |
| **Cost Analysis** | | |  | | | |
| Difference in Mean Total Cost €  (95% CIs) [p-value] | | | **-2641**  **(-6467, 1186) [0.1720]** | | | |
| **Effectiveness Analysis** | | |  | | | |
| Difference in Mean QALYs  (95% CIs) [p-value] | | | 0.059  (-0.012, 0.129) [0.101] | | | |
|  | | | | | | |
| ***Probability (%) that the Intervention is Cost Effective for Threshold Value (λ)*** | | | | | | |
| **λ = €0** | **λ = €5,000** | **λ = €10,000** | | **λ = €20,000** | **λ = €30,000** | **λ = €45,000** |
| 0.904 | 0.919 | 0.928 | | 0.938 | 0.944 | 0.952 |
| **Variable/ Analysis** | | | **Subgroup Analysis: < 4 Chronic Conditions (n=53)** | | | |
| **Cost Analysis** | | |  | | | |
| Difference in Mean Total Cost €  (95% CIs) [p-value] | | | **-389**  **(-3952, 3174) [0.826]** | | | |
| **Effectiveness Analysis** | | |  | | | |
| Difference in Mean QALYs  (95% CIs) [p-value] | | | 0.001  (-0.107, 0.107) [0.990] | | | |
|  | | | | | | |
| ***Probability (%) that the Intervention is Cost Effective for Threshold Value (λ)*** | | | | | | |
| **λ = €0** | **λ = €5,000** | **λ = €10,000** | | **λ = €20,000** | **λ = €30,000** | **λ = €45,000** |
| 0.542 | 0.574 | 0.609 | | 0.681 | 0.732 | 0.789 |

**Cost Analyses**: *Independent t test*

**QALYs Analyses**: *Independent t test*

**Expected Cost Effectiveness Analysis**: Probabilities estimated using nonparametric bootstrapping technique based on 1000 bootstrapped resamples.

**Note:** Data were collected during the study period from November 2015 to December 2018

**Figure A1 – Cost Effectiveness Acceptability Curve**
